# Transfer factor for carbon monoxide in patients with COPD and diabetes: results from the German COSYCONET cohort

**DOI:** 10.1186/s12931-016-0499-0

**Published:** 2017-01-13

**Authors:** Kathrin Kahnert, Tanja Lucke, Frank Biertz, Andreas Lechner, Henrik Watz, Peter Alter, Robert Bals, Jürgen Behr, Rolf Holle, Rudolf M. Huber, Stefan Karrasch, Beate Stubbe, Margarethe Wacker, Sandra Söhler, Emiel F. M. Wouters, Claus Vogelmeier, Rudolf A. Jörres, for the COSYCONET study group

**Affiliations:** 1 Department of Internal Medicine V, University of Munich, Comprehensive Pneumology Center, Member of the German Center for Lung Research, Ziemssenstr. 1, 80336 Munich, Germany; 2 Institute and Outpatient Clinic for Occupational, Social and Environmental Medicine, Ludwig-Maximilians-Universität München, Ziemssenstr. 1, 80336 Munich, Germany; 3 Institute for Biostatistics, Hannover Medical School, Carl-Neuberg-Str. 1, 30625 Hannover, Germany; 4 Department of Internal Medicine IV, University of Munich, Ziemssenstr. 1, 80336 Munich, Germany; 5 Pulmonary Research Institute at LungenClinic Grosshansdorf, Airway Research Center North, Member of the German Center for Lung Research, Woehrendamm 80, 22927 Grosshansdorf, Germany; 6 Department of Respiratory Medicine, University of Marburg, University Giessen and Marburg Lung Center (UGMLC), Member of the German Center for Lung Research, Baldingerstraße, 35043 Marburg, Germany; 7 Department of Internal Medicine V — Pulmonology, Allergology, Respiratory Intensive Care Medicine, Saarland University Hospital, Kirrberger Straße 1, 66424 Homburg, Germany; 8 Institute of Health Economics and Health Care Management, Helmholtz Zentrum München (GmbH) — German Research Center for Environmental Health, Member of the German Center for Lung Research, Comprehensive Pneumology Center Munich (CPC-M), Ingolstaedter Landstr. 1, 85764 Neuherberg, Germany; 9 Department of Internal Medicine B — Cardiology, Intensive Care, Pulmonary Medicine and Infectious Diseases, Scientific Division of Pneumology and Pneumological Epidemiology, University Medicine Greifswald, Ferdinand-Sauerbruch-Strasse, 17475 Greifswald, Germany; 10 ASCONET Study Coordination Office, University of Marburg, Baldingerstraße, 35043 Marburg, Germany; 11 Department of Respiratory Medicine, Maastricht University Medical Center, P. Debyelaan 25, 6202 AZ Maastricht, The Netherlands; 12 Institute of Epidemiology I, Helmholtz Zentrum München — German Research Center for Environmental Health, Ingolstaedter Landstr. 1, 85764 Neuherberg, Germany

**Keywords:** COPD, Diabetes, Lung function, Diffusing capacity

## Abstract

**Background:**

An impairment of CO diffusing capacity has been shown in diabetic patients without lung disease. We analyzed how diffusing capacity in patients with COPD is affected by the concurrent diagnosis of diabetes.

**Methods:**

Data from the initial visit of the German COPD cohort COSYCONET were used for analysis. 2575 patients with complete lung function data were included, among them 358 defined as diabetics with a reported physician diagnosis of diabetes and/or specific medication. Pairwise comparisons between groups and multivariate regression models were used to identify variables predicting the CO transfer factor (TLCO%pred) and the transfer coefficient (KCO%pred).

**Results:**

COPD patients with diabetes differed from those without diabetes regarding lung function, anthropometric, clinical and laboratory parameters. Moreover, gender was an important covariate. After correction for lung function, gender and body mass index (BMI), TLCO%pred did not significantly differ between patients with and without diabetes. The results for the transfer coefficient KCO were similar, demonstrating an important role of the confounding factors RV%pred, TLC%pred, ITGV%pred, FEV_1_%pred, FEV_1_/FVC, age, packyears, creatinine and BMI. There was not even a tendency towards lower values in diabetes.

**Conclusion:**

The analysis of data from a COPD cohort showed no significant differences of CO transport parameters between COPD patients with and without diabetes, if BMI, gender and the reduction in lung volumes were taken into account. This result is in contrast to observations in lung-healthy subjects with diabetes and raises the question which factors, among them potential anti-inflammatory effects of anti-diabetes medication are responsible for this finding.

## Background

Patients with chronic obstructive pulmonary disease (COPD) show a high number of comorbidities. One of the frequent comorbidities is diabetes mellitus, which is of particular relevance through its association with cardiovascular diseases. There is evidence that lung emphysema, a frequent phenotype in COPD, is initiated by changes of the alveolar-capillary system [1]. On the other hand, diabetes is known to induce micro— and macroangiopathy [2], with microangiopathy causing nephropathy, retinopathy or neuropathy, and macroangiopathy contributing to the development of myocardial infarction, stroke and gangrene [3]. Impaired capillary function may have a negative impact on alveolar gas exchange. This raises the question whether the vascular alterations associated with diabetes interact with those of COPD.

A clinically established measure of pulmonary capillary function is the diffusing capacity for carbon monoxide (CO) which is closely linked to the degree of lung emphysema as quantified by CT scans [4]. On the other hand, patients with diabetes without known lung disease also may show a decreased CO diffusing capacity [5, 6], and impairment of pulmonary function in type I diabetes patients treated with insulin was linked to the quality of metabolic control [7]. The combined effects of COPD and diabetes on the CO diffusing capacity are unclear. In patients with COPD and diabetes only studies on spirometry are available [8], whereas the CO transfer factor has been measured only in diabetic patients without COPD. These considerations suggest a link between diabetes, emphysema and reduced diffusing capacity, but diabetes has also been associated with predominant airway obstruction [9] which would imply a higher but not lower CO diffusing capacity relative to obstruction.

Based on the assumption that vascular alterations arising from diabetes could modify the reduction of CO diffusing capacity typically found in COPD, we analyzed the data of the *German* ***CO*** *PD and* ***Sy*** *stemic Consequences-* ***Co*** *morbidities* ***Net*** *work (COSYCONET).* This is a multi-center cohort study investigating the relationship of COPD with comorbidities. It includes 2741 patients of age ≥ 40 years with diagnosis of COPD, among them 376 patients with the reported comorbidity of diabetes. The hypothesis underlying our analysis was that the changes of CO diffusing capacity associated with diabetes and COPD add to each other reflecting the additional impairment of the capillary status due to diabetes. For this purpose we evaluated lung function, clinical and anthropometric data as well as biomarkers including HbA1c by multivariate regression analyses that took into account the factors influencing CO diffusing capacity, in terms of total lung transfer factor (TLCO) and volume-related transfer coefficient (KCO).

## Methods

### Patients

This study analyzed data of the initial visit of the German COPD cohort COSYCONET. The cohort was recruited through the outpatient and inpatient sector, patients’ organizations and media campaigns. Patients with previous lung transplantation, lung volume reduction surgery or lung malignancies were not eligible, as well as patients with moderate or severe exacerbations within four weeks prior to the visit. In the time period between September 2010 and December 2013 patients were examined in 31 study centers. The characteristics of the cohort have been described elsewhere [10, 11].

### Assessments

The patients’ clinical and functional state was assessed by a wide spectrum of tests, with focus on pulmonary function and cardiovascular comorbidities. The assessments included a detailed history regarding concomitant diseases and regularly taken medication as well as the evaluation of biomarkers in blood and were guided by SOPs following established guidelines [10]. Spirometric parameters comprised forced expiratory volume in one second (FEV_1_), forced vital capacity (FVC) and their ratio (FEV_1_/FVC); bodyplethysmographic parameters included intrathoracic gas volume (ITGV) and total lung capacity (TLC). The diffusing capacity for carbon monoxide (CO) was determined using the single-breath technique. This measurement yielded the transfer factor for CO (TLCO) and the transfer coefficient (KCO) as the ratio of TLCO and alveolar volume (VA). Predicted values for spirometry were taken from the Global Lung Function Initiative (GLI) data [12], those for bodyplethysmography from Koch et al. [13], except for ITGV [14], and those for TLCO and KCO from van der Lee et al. [15].

### Diagnosis of COPD and diabetes

The diagnosis of COPD was based on lung function impairment according to GOLD criteria [16]. In addition to GOLD 1–4, patients of the former category GOLD 0 were included, i.e. patients with normal lung function according to the GOLD criteria but with symptoms of chronic bronchitis and a smoking history [10]. Only patients with valid lung function data were included (*n* = 2,575) among whom 349 reported diabetes as diagnosed by a physician. Patients were grouped into four categories depending on the matching between the patients’ report and either disease-specific or non-specific but disease-compatible medication: there were 268 patients with reported diabetes and diabetes-specific medication (A), 9 patients with diabetes-specific medication in the absence of a reported diagnosis (B). 24 patients took medication that was compatible with but not specific for diabetes (C), and 57 took no diabetes-related medication at all (D) [17]. In the present analysis we defined diabetes by self-reported physician-based diagnosis irrespective of medication, or the intake of diabetes-specific medication in the absence of a reported diagnosis (*n* = 358; extended definition: A + B + C + D). For control purposes the results were compared with those obtained in patients defined by the presence of diabetes-specific medication only (*n* = 277; restrictive definition: A + B).

### Statistical analysis

For data description mean values and standard deviations (SD) were used. The groups with and without diabetes were compared in a first step by unpaired *t*-test, categorical data were analyzed by the chi-square-test. As some variables showed deviations from normal distribution according to the Kolmogorov-Smirnov-test, we additionally used the Mann–Whitney-*U*-test as a non-parametric method to check the results. In a second step multivariate linear regression analyses were performed, or analysis of covariance (ANCOVA), with TLCO or KCO in %predicted taken as dependent measures and the binary categories diabetes and gender as independent factors, in addition to covariates. The following covariates were considered and eventually removed from the models in a stepwise backward elimination fashion: FEV_1_%pred, FEV_1_/FVC, sRaw (specific airway resistance), TLC%pred, BMI, packyears, hemoglobin (Hb) and HbA1c. The level of statistical significance was set at *p* = 0.05 and the statistical analyses were performed by SPSS Statistics 23 (IBM Corp., Armonk, NY, USA).

## Results

### Comparison of diabetes and non-diabetes group

Clinical and anthropometric characteristics are given in Table 1, diabetes being defined either by the extended or the restrictive definition (see methods).

**Table 1:** Baseline characteristics of the subgroups with and without diabetes

Parameter	Non-diabetes patients	Diabetes patients (extended def.)	*p*-values (extended def.)	Diabetes patients (restrictive def.)	*p*-values (restrictive def.)
Gender (m/f)	1270/947(57/43%)	262/96 (73/27%)	*p* < 0.001*	208/69 (76/24%)	*p* < 0.001*
Age (y)	64.7 (±8.7)	67.0 (±7.6)	*p* < 0.001*	67.1 (±7.5)	*p* < 0.001*
BMI (kg/m^2^)	26.4 (±5.05)	30.5 (±5.8)	*p* < 0.001*	30.69 (±5.1)	*p* < 0.001*
Waist circumf. (cm)	97.7 (±15.1)	110.0 (±14.7)	*p* < 0.001*	111.1 (±14.3)	*p* < 0.001*
Packyears	46.8 (±34.8)	55.3 (±40.9)	*p* < 0.001*	55.4 (±40.5)	*p* < 0.001*
Hb (mg/dl)	14.66 (±1.38)	14.43 (±1.39)	*p* = 0.003*	14.46 (±1.32)	*p* = 0.032*
HbA1c (%)	5.76 (±0.47)	6.91 (±1.0)	*p* < 0.001*	7.03(±1.13)	*p* < 0.001*
Creatinine (mg/dl)	0.88 (±0.25)	0.96 (±0.29)	*p* < 0.001*	0.95 (±0.27)	*p* < 0.001*
FEV_1_%pred	59.7 (±22.2)	59.8 (±21.2)	*p* = 0.906	59.6 (±20.0)	*p* = 0.973
FEV_1_/FVC	54.8 (±13.6)	55.3 (±13.2)	*p* = 0.431	54.5 (±13.0)	*p* = 0.687
FVC%pred	78.6 (±19.1)	79.1 (±19.1)	*p* = 0.638	78.0 (±18.7)	*p* = 0.223
TLC%pred	112.9 (±30.4)	107.2 (±27.6)	*p* < 0.001*	106.3(±27.3)	*p* < 0.001*
RV%pred	150.1 (±47.2)	143.4 (±45.2)	*p* = 0.012*	142.4 (±43.5)	*p* ≤ 0.011*
ITGV%pred	146.6 (±36,8)	135.1 (±36.0)	*p* < 0.001*	134.5 (±35.3)	*p* < 0.001*
TLCO%pred	52.8 (±21.0)	57.1 (±19.6)	*p* < 0.001*	57.0 (±19.2)	*p* = 0.001*
KCO%pred	66.1 (±23.8)	75.6 (±23.4)	*p* < 0.001*	76.3 (±23.6)	*p* < 0.001*
GOLD 0/1/2/3/4	269/242/868/679/159 (12/11/39/31/7%)	68/33/133/103/21 (19/9/37/29/6%)	*p* = 0.010*	51/26/107/78/15 (18/9/39/28/5%)	*p* = 0.066
GOLD A/B/C/D	239/1198/32/735 (11/54/2/33%)	37/170/12/138 (10/48/3/39%)	*p* = 0.008*	32/130/10/104 (12/47/4/38%)	*p* = 0.017*

The table shows mean values and standard deviations or absolute numbers, in case of gender and COPD classes additionally percentages (deviations of total from 100% are due to rounding). Column 3 shows the results of comparisons between the diabetes group (extended definition) and the complementary group of non-diabetes patients. Column 5 shows the corresponding results of comparisons between diabetes group (restrictive definition) and the corresponding group of non-diabetes patients. The comparisions between groups were performed by unpaired *t*-tests, either for equal or unequal variances depending on the data, or by chi-square-tests in the case of categorical variables

Significant (*p* < 0.05) differences are marked with (*)

When comparing the mean values between patients with and without diabetes, gender, age, BMI, waist circumference, packyears, hemoglobin, HbA1c, creatinine, TLC%pred, RV%pred, ITGV%pred, TLCO%pred, KCO%pred and the distribution of GOLD stages, both 1–4 and A-D, turned out to be significantly different (*p* ≤ 0.01 each). The results for the restrictive definition of diabetes were similar, but the distribution of GOLD stages 1–4 did not significantly differ, in contrast to GOLD A-D (*p* = 0.017). Moreover the non-parametric testing yielded the same distribution of significance across variables as the parametric testing.

In patients with diabetes, the prevalence of reported comorbidities was significantly higher for arterial hypertension, myocardial infarction, coronary heart disease, cardiac arrhythmia, dyslipidemia, peripheral neuropathy, gastrointestinal disorders, hyperuricemia (*p* < 0.001 each) and osteoporosis (*p* = 0.047). The proportion of patients with asthma did not significantly differ between both groups.

### Gender and diabetes

As the gender distribution differed between diabetic and non-diabetic patients, we stratified the parameters given in Table 1 according to gender; the results are given in Table 2.

**Table 2:** Comparison of subgroups stratified according to diabetes and gender

Parameter	Male patients(all)	Male/non-diabetes	Male/diabetes extended def.	Female patients(all)	Female/non-diabetes	Female/diabetes extended def.
Age (y)	65.8 ± 8.5	65.5 ± 8.6	67.1 ± 7.6°	63.8 ± 8.7*	63.5 ± 8.7*	66.6 ± 7.4°
BMI (kg/m^2^)	27.4 ± 4.9	26.9 ± 4.6	30.1 ± 5.5°	26.2 ± 5.8*	25.7 ± 5.5*	31.6 ± 6.5*°
Waist circumf. (cm)	104.6 ± 13.6	103.2 ± 13.0	111.4 ± 14.2°	91.6 ± 15.2*	90.2 ± 14. 4*	105.8 ± 15.4*°
Packyears	54.0 ± 38.8	52.1 ± 38.0	57.2 ± 42.4	40.2 ± 28.7*	39.8 ± 28.6*	48.8 ± 34.7°
Hb (mg/dl)	15.02 ± 1.38	15.09 ± 1.38	14.66 ± 1.33°	14.06 ± 1.16*	14.09 ± 1.13*	13.79 ± 1.36*°
HbA1c (%)	5.98 ± 0.76	5.78 ± 0.48	6.94 ± 1.09°	5.82 ± 0.64*	5.72 ± 0.45*	6.85 ± 1.15°
Creatinine (mg/dl)	0.97 ± 0.24	0.96 ± 0.23	1.00 ± 0.30°	0.78 ± 0.23*	0.77 ± 0.23*	0.84 ± 0.21*°
FEV_1_%pred	58.0 ± 21.7	58.0 ± 21.9	58.1 ± 20.3	62.2 ± 22.3*	61.9 ± 22.3*	64.6 ± 22.5*
FEV_1_/FVC	54.5 ± 13.5	54.5 ± 13.5	54.8 ± 13.2	55.1 ± 13.7	55.0 ± 13.7	56.9 ± 13.2
FVC%pred	79.0 ± 18.8	79.0 ± 18.9	79.4 ± 18.7	78.0 ± 19.4	78.0 ± 19.3	78.3 ± 20.3
TLC%pred	100.0 ± 24.2	100.0 ± 24.4	100.0 ± 23.4	129.9 ± 29.1*	130.1 ± 29.1*	127.6 ± 28.6*
RV%pred	149.5 ± 48.1	150.7 ± 48.8	142.2 ± 42.0°	144.4 ± 52.9	149.5 ± 45.0	144.0 ± 47.9
ITGV%pred	141.4 ± 36.2	142.9 ± 36.2	134.0 ± 34.9°	150.3 ± 37.4*	151.5 ± 36.6*	138.0 ± 40.2°
TLCO%pred	55.2 ± 21.0	54.6 ± 21.4	58.0 ± 19.0°	50.6 ± 20.4*	50.3 ± 20.2*	54.6 ± 21.1°
KCO%pred	70.6 ± 24.3	69.4 ± 24.5	77.1 ± 22.7°	62.5 ± 22.6*	61.6 ± 22.2*	71.1 ± 25.0*°
GOLD 0/1/2/3/4	166/159/597/484/126	126/137/494/403/110	40/22/103/81/16	171/116/404/298/54*	143/105/374/276/49*	28/11/30/22/5*°

The table shows mean values and standard deviations or absolute numbers. Column 2 and 5 show the results of comparisons between females and males irrespective of diabetes, significant (*p* < 0.05) differences are marked with (*). Column 3 and 6 show the results of comparisons between females and males without diabetes, significant differences are marked with (*). Column 4 and 7 show the results of comparisons between male and female diabetes patients, significant differences are marked with (*). Column 3 and 4 shows the results of the comparison between male non-diabetes and male diabetes patients, significant (*p* < 0.05) differences are marked with (°). Column 6 and 7 show the comparison between female non-diabetes and female diabetes patients, significant differences are marked with (°). The comparisons between groups were performed by unpaired t-tests, either for equal or unequal variances depending on the data, or by chi-square-tests in the case of categorical variables

When comparing male and female patients irrespective of diabetes, most parameters were significantly different from each other (*p* < 0.05), in particular those of CO diffusing capacity and FEV_1_%pred. The comparison within non-diabetic patients revealed a similar pattern of differences between males and females, including KCO%pred and FEV_1_%pred. Patients with diabetes showed a lower number of gender-related significant differences, including FEV_1_%pred but not TLCO%pred. The distribution of GOLD stages also differed between males and females, and this was true for the total group of patients (*p* < 0.001), as well as non-diabetes (*p* < 0.001) and diabetes patients (*p* = 0.030). With non-parametric testing the distribution of significances across variables remained the same except for KCO%pred in diabetes patients, which became non-significant.

Moreover Table 2 presents the results of comparisons between diabetic and non-diabetic patients for males and females separately. In males, age, BMI, waist circumference, hemoglobin, HbA1c and creatinine significantly differed between both groups, as well as RV%pred, ITGV%pred, TLCO%pred and KCO%pred (*p* < 0.05 each). In females, age, BMI, waist circumference, packyears, hemoglobin, HbA1c and creatinine differed between the two groups, as well as ITGV%pred, KCO%pred and TLCO%pred (*p* < 0.05 each). Non-parametric testing showed only for male patients a difference from parametric testing regarding creatinine which became non-significant.

### Diffusing capacity versus functional/clinical parameters

**Fig. 1:**
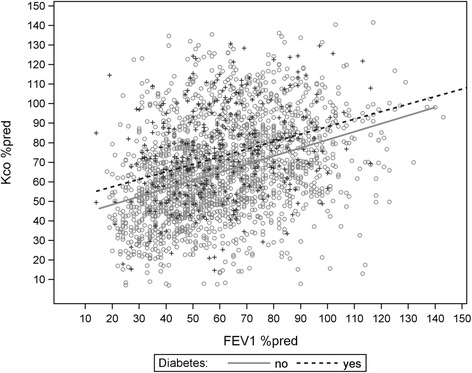
Relationship between KCO%pred and FEV_1_%pred. The regression lines refer to KCO%pred adjusted for FEV_1_%pred, TLC%pred, ITGV%pred, RV%pred, FEV_1_/FVC, packyears, age, gender, BMI, HbA1c (%) and creatinine. There was no significant difference between diabetic and non-diabetic patients (extended definition)

For KCO%pred as dependent variable, multivariate linear regression analyses using as independent variables the parameters listed in Table 1 plus diabetes and backward selection, yielded gender, age, BMI, packyears, FEV_1_%pred, FEV_1_/FVC, TLC%pred, RV%pred, ITGV%pred (*p* ≤ 0.001 each) and creatinine (*p* = 0.033) as significant predictors (Table 3). A similar analysis for TLCO%pred yielded gender, BMI, packyears, FEV_1_%pred, RV%pred, ITGV%pred and HbA1c (*p* ≤ 0.004 each) and creatinine (*p* = 0.035) (data not shown). In both analyses, diabetes was no significant predictor, neither as extended nor as restrictive definition. The relationship between KCO%pred, FEV_1_%pred and diabetes is illustrated in Fig. 1 using adjusted values. It demonstrates the overlap between the two groups and the fact that there is no tendency towards lower values in diabetes patients after adjustment. When using the GOLD A-D categorization in addition to FEV_1_ and diabetes as predictors of KCO%pred, the GOLD categories turned out to be not significantly associated with KCO%pred. The same was true for TLCO%pred.

**Table 3:** Multivariate regression analysis with KCO%pred as dependent variable

Modell	Non-standardized coefficients		Standardized coefficients	T	*p*-value
	Regression coefficient B	Standard error	Beta		
Constant	47.707	6.378		7.480	.000
Gender (m = 1.f = 2)	−5.810	1.160	-.121	−5.008	.000
Age (y)	.342	.056	.123	6.150	.000
BMI (kg/m^2^)	1.034	.092	.237	11.256	.000
Packyears	-.059	.012	-.091	−4.894	.000
FEV_1_%pred	.282	.025	.257	11.140	.000
FEV_1_/FVC	-.113	.034	-.066	−3.346	.001
TLC%pred	-.070	.018	-.089	−3.883	.000
RV%pred	.112	.022	.221	5.127	.000
ITGV%pred	-.253	.028	-.393	−9.121	.000
Creatinine (mg/dl)	−4.253	1.991	-.044	−2.136	.033

### Lung function parameters

CO transfer factor and coefficient differed between diabetes and non-diabetes groups, if considered without adjustment for other variables (Tables 1 and 2). The adjustment via multivariate regression analysis showed the difference in KCO%pred to be explained by a number of confounders, among them parameters of airway obstruction and lung hyperinflation. In order to reveal how much had to be attributed to these parameters, we repeated the regression analyses using only lung function parameters as independent variables. FEV_1_%pred, FEV_1_/FVC, TLC%pred, RV%pred, ITGV%pred, as well as gender and diabetes turned out to be significant predictors of KCO%pred (*p* ≤ 0.008 each). Analysis of covariance (ANCOVA) showed that there was no significant interaction term between gender and diabetes. In case of TLCO%pred, regression analysis revealed that FEV_1_%pred, RV%pred, ITGV%pred and gender were significant predictors (*p* < 0.001 each) while diabetes was borderline non-significant (*p* = 0.056). When using the restrictive definition of diabetes, the results were qualitatively similar.

To reveal to which extent the other parameters were important in the adjustment of KCO%pred, we added each of these to lung function as predictors in the multivariate regression analysis. It turned out that BMI but not age, packyears or creatinine eliminated (*p* = 0.079) the significant difference between diabetes and non-diabetes that was seen with lung function parameters only. In a similar manner, for TLCO%pred the introduction of BMI eliminated (*p* = 0.918) the borderline effect of diabetes. Thus BMI appeared to be a decisive factor for the differences in CO diffusing capacity between diabetes and non-diabetes.

## Discussion

The analysis of data from a large COPD cohort showed that there was no significant difference of CO transport parameters, especially TLCO%pred and KCO%pred, between patients with COPD and diabetes compared to non-diabetes COPD patients, provided that lung volumes, BMI and gender were taken into account as confounders. In particular, diabetes was associated with a reduction in lung volume and an increase in BMI. CO diffusing capacity was not deteriorated by the presence of diabetes. Instead, after adjustment for other parameters there was still a tendency towards better KCO values in diabetes. This finding differs from observations in diabetes patients without COPD who showed a slightly impaired CO diffusing capacity.

Our study population comprised all patients with complete lung function data and severity GOLD ≥0 from the COSYCONET cohort [18]. This cohort is particularly suited for investigating the relationship between comorbidities and functional status [10]. The information on medication allowed the definition of diabetes in terms of self-reported physician-based diagnosis and/or diabetes-specific medication; patients had been asked to bring all their medication to the study center [10]. The extended definition was based on self-reported diagnosis and/or the presence of specific medication, the restrictive definition on the presence of diabetes-specific medication only and was used for sensitivity analyses [17].

CO diffusing capacity is established in the evaluation COPD and lung emphysema [5, 19]. In diabetes patients with or without poor glycemic control or microangiopathic complications, but without lung disease, studies revealed a reduction of CO diffusing capacity and a restrictive pattern of spirometric parameters [6, 18, 20], but no correlation with the duration of diabetes [20]. The potential role of obesity was addressed by comparing diabetes with obese non-diabetes patients [21]; TLCO was reduced in diabetes. In addition, diabetic neuropathy, macrovascular complications, impaired renal function and insulin treatment were linked to low TLCO [21]. A meta-analysis summarized the association between diabetes and a restrictive lung function pattern in terms of FEV_1_, FVC and CO diffusing capacity, irrespective of BMI, smoking, diabetes duration and HbA1c levels in lung-healthy subjects [22]. There are very few studies in patients with COPD and diabetes. Among diabetes patients with and without COPD only those from an “unclassified” sub-group according to standard GOLD categories showed a reduction of FEV_1_%pred and FVC%pred [8]. Surprisingly, CO diffusing capacity has not been studied in patients with COPD and diabetes.

The presence of diabetes in COPD was associated with impairments of CO diffusing capacity beyond those attributable to COPD. On average, diabetes patients were older (Table 1) and more often males compared to non-diabetes patients (75% in diabetes, 58% in the total cohort). Diabetic patients showed higher BMI, waist circumference, serum HbA1c and creatinine, and lower hemoglobin levels. They reported more packyears and cardiovascular comorbidities. The unadjusted mean values of lung function including CO diffusing capacity, differed between diabetic and non-diabetic patients. To account for the effect of gender, we stratified but many of the differences between diabetes and non-diabetes remained significant (Table 2). Obviously, gender was not responsible for the differences between the two groups, particularly regarding KCO%pred.

To identify confounders we performed multivariate regression analyses. Both KCO (Table 3) and TLCO depended on other lung function variables, laboratory parameters and gender, without significant effect of diabetes. We then reduced the set of predictors to lung function and gender, to reveal whether lung function was the major confounder. There was again a significant dependence on diabetes regarding KCO%pred but not TLCO%pred. Accounting for age, packyears or creatinine did not eliminate the difference of KCO%pred between non-diabetes and diabetes. When BMI and gender were chosen as predictors, the effect of diabetes became non-significant (*p* = 0.086); the use of BMI, lung function and gender as predictors completely abolished the effect of diabetes. Therefore, besides FEV_1_%pred, the major factor explaining the difference of KCO%pred between non-diabetes and diabetes appeared to be BMI. This is reflected in the large regression coefficients of BMI and FEV_1_%pred in Table 3. Waist circumference did not yield as conclusive results as BMI; it was eliminated in most regression analyses, particularly in competition with BMI. To address the question whether the degree of glycemic control affected the result, we repeated the multivariate regression analyses using insulin-monotherapy (*n* = 65) or the presence of HbA1c values ≥8% (*n* = 46) as additional predictors. None of them emerged as significant regarding KCO and TLCO (data not shown). Patients of the restrictive definition showed higher levels of HbA1c and presumably had poorer glycemic control. However, the results regarding CO diffusing capacity essentially did not depend on the definition chosen.

These observations suggest that microangiopathic alterations caused by diabetes did not manifest as additional pulmonary vascular changes detectable through impaired CO diffusing capacity in COPD. The finding that the unadjusted transfer coefficient KCO was higher (by nearly 10%) in diabetes may be due to the reduction in lung volume and increase in BMI. Accordingly, the values of TLCO, a parameter which takes into account lung volume, were more similar, TLCO%pred being larger by only about 5% in diabetes. These findings suggest that in diabetes patients with COPD a reduction in lung volume and a higher BMI were responsible for the relatively increased values of unadjusted KCO%pred.

A recent radiological study reported diabetes as a risk factor for obstructive airway disease but not emphysema [9]. This observation is in accordance with our findings insofar as reductions in CO diffusing capacity are more common in emphysema than obstructive airway disease per se. In view of the microvascular defects associated with diabetes [23], it seems counterintuitive to link diabetes to airway obstruction rather than to an intrinsically vascular disease such as emphysema. From this point of view it seems remarkable that in our study the diabetes patients, at least on average, did not even show a tendency towards an impairment of CO diffusing capacity parameters, despite the fact that they reported a higher number of packyears and comorbidities, including cardiovascular diseases. This could be relevant as e.g. heart failure has a negative impact on CO diffusing capacity [24]. It might well be that the changes in pulmonary capillary bed associated with lung emphysema are dominant over microvascular impairments associated with diabetes.

Noteworthy, systemic anti-inflammatory effects of anti-diabetic medication are increasingly considered [25], such as metformin [26, 27], glucagon-like peptide-1 analogs [28], sulfonylureas [29], thiazolidinedione, DPP-4 inhibitors [30] and insulin [31]; all of them are inhibitors of NF-κB which plays a central role in COPD-associated inflammation [32]. In non-diabetic patients metformin did not ameliorate acute exacerbations [33], but exerted positive effects on symptoms, health status, inspiratory muscle function, lung hyperinflation and gas trapping in a prospective open-label study of moderate to severe COPD patients with diabetes and BMI > 25 kg/m^2^ [34]. Possibly the beneficial effects of anti-diabetic drugs on the lung occur only in the presence of definite inflammatory lung disease such as COPD.

In our study, most diabetes patients took medications of the type mentioned (*n* = 147 metformin monotherapy, *n* = 65 insulin monotherapy; *n* = 202 metformin combined with other oral specific medication, *n* = 31 plus insulin, *n* = 12 glinides, *n* = 23 DPP4I, *n* = 50 sulfonylureas, *n* = 7 incretin mimetics (non-exclusive groups)). Accordingly, it was not possible to define a sufficiently large control group of diabetes patients without such medication. Based on our data we cannot explain the reported impairment of CO diffusing capacity in non-COPD diabetes patients. Such a reduction might be derived from both a slight reduction of lung volume as an “external” factor and alterations of the pulmonary vascular bed as an “internal” factor reflecting morphological alterations in which inflammation plays a role. Both hyper- and hypoglycemic states have been reported to be associated with pro-inflammatory effect [35, 36] which theoretically could affect the lung. On the other hand it does not seem implausible in COPD patients the inflammatory part involved in the reduction of CO diffusing capacity is dominant over the diabetes part and more effectively targeted by the anti-inflammatory effects of the diabetes medication.

### Limitations of the study

We could statistically adjust for a number of factors but mostly without interaction terms which would have required an even greater sample size. In addition the analysis was influenced by the unequal distribution of males and females across the two groups. This was relevant as even in the group without diabetes the parameters of CO diffusing capacity were gender-dependent. Information on the type and duration of diabetes was not available. The extended definition represented patients with lower HbA1c levels and probably better glycemic control compared to patients obeying the restrictive definition. A further grouping into patients with very poor glycemic control, e.g. HbA1c values ≥8% or insulin-monotherapy, did not yield conclusive results, probably due to the small sample sizes associated with these requirements. The statistical analyses indicated that the diabetes definition was not critical for the major finding, therefore the identification of diabetes patients was probably not a limiting factor in the study. A further limitation is that a detailed analysis of comorbidities was not possible due to the relatively small numbers that remained, particularly for their combinations, in the diabetes group. Moreover these comorbidities could not be verified by specific medication to the same extent as diabetes.

## Conclusion

The analysis of data from a large COPD cohort showed that the transfer factor TLCO in terms of %predicted did not significantly differ between patients with and without diabetes, if other differences, especially those of lung function and body weight, were taken into account. The findings for the volume-related transfer coefficient KCO were similar but demonstrated the role of confounding factors such as lung volume and BMI with even greater clarity. Former investigations in diabetes patients without lung disease showed an impairment of CO diffusing capacity. Therefore, intuitively one might expect an additional reduction of diffusing capacity in COPD patients with diabetes and therefore be inclined to attribute a low value at least partially to diabetes. As a clinical implication, our study indicates that a reduction of diffusing capacity must be fully attributed to the lung disease and not to diabetes. This contrasts with other comorbidities, such as anemia which directly affects diffusing capacity. The question which factors may be responsible for our observations, remains open: either microvascular alterations caused by diabetes are not relevant in a lung disease such as COPD, or the common anti-diabetic drugs exert a beneficial, anti-inflammatory effect.

## Abbreviations

%pred: Percent predicted
ANCOVA: Analysis of covariance
BMI: Body mass index
CO: Carbon monoxide
COPD: Chronic obstructive pulmonary disease
FEV_1_: Forced expiratory volume in one second
FVC: Forced vital capacity
Hb: Hemoglobin
HbA1c: Hemoglobin A1c
ITGV: Intrathoracic gas volume
KCO: CO transfer coefficient
SOP: Standard operating procedure
sRaw: Specific airway resistance
TLC: Total lung capacity
TLCO: CO transfer factor
VA: Alveolar volume

## References

[1] Voelkel NF, Gomez-Arroyo J, Mizuno S (2011). COPD/emphysema: the vascular story. Pulm Circ.

[2] Katakura M, Naka M, Kondo T, Komatsu M, Yamauchi K, Hashizume K, Aizawa T (2007). Development, worsening, and improvement of diabetic microangiopathy in older people: six-year prospective study of patients under intensive diabetes control. J Am Geriatr Soc.

[3] Birrer M (2001). Macroangiopathy in diabetes mellitus. Vasa.

[4] Choromanska A, Macura KJ (2012). Role of computed tomography in quantitative assessment of emphysema. Pol J Radiol.

[5] Weinreich UM, Thomsen LP, Brock C, Karbing DS, Rees SE (2015). Diffusion capacity of the lung for carbon monoxide — a potential marker of impaired gas exchange or of systemic deconditioning in chronic obstructive lung disease?. Chron Respir Dis.

[6] Uz-Zaman S, Banerjee J, Singhamahapatra A, Dey PK, Roy A, Roy K, Roy Basu K (2014). Assessment of lung function by spirometry and diffusion study and effect of glycemic control on pulmonary function in type 2 diabetes mellitus patients of the eastern India. J Clin Diagn Res.

[7] Dieterle CD, Schmauss S, Arbogast H, Domsch C, Huber RM, Landgraf R (2007). Pulmonary function in patients with type 1 diabetes before and after simultaneous pancreas and kidney transplantation. Transplantation.

[8] Kinney GL, Black-Shinn JL, Wan ES, Make B, Regan E, Lutz S, Soler X, Silverman EK, Crapo J, Hokanson JE (2014). Pulmonary function reduction in diabetes with and without chronic obstructive pulmonary disease. Diabetes Care.

[9] Hersh CP, Make BJ, Lynch DA, Barr RG, Bowler RP, Calverley PM, Castaldi PJ, Cho MH, Coxson HO, DeMeo DL (2014). Non-emphysematous chronic obstructive pulmonary disease is associated with diabetes mellitus. BMC Pulm Med.

[10] Karch A, Vogelmeier C, Welte T, Bals R, Kauczor HU, Biederer J, Heinrich J, Schulz H, Glaser S, Holle R (2016). The German COPD cohort COSYCONET: Aims, methods and descriptive analysis of the study population at baseline. Respir Med.

[11] Jorres RA, Welte T, Bals R, Koch A, Schnoor M, Vogelmeier C (2010). Systemic manifestations and comorbidities in patients with chronic obstructive pulmonary disease (COPD) and their effect on clinical state and course of the disease--an overview of the cohort study COSYCONET. Dtsch Med Wochenschr.

[12] Quanjer PH, Stanojevic S, Cole TJ, Baur X, Hall GL, Culver BH, Enright PL, Hankinson JL, Ip MS, Zheng J (2012). Multi-ethnic reference values for spirometry for the 3-95-yr age range: the global lung function 2012 equations. Eur Respir J.

[13] Koch B, Friedrich N, Volzke H, Jorres RA, Felix SB, Ewert R, Schaper C, Glaser S (2013). Static lung volumes and airway resistance reference values in healthy adults. Respirology.

[14] Quanjer PH, Tammeling GJ, Cotes JE, Pedersen OF, Peslin R, Yernault JC (1993). Lung volumes and forced ventilatory flows. Eur Respir J.

[15] van der Lee I, Zanen P, Stigter N, van den Bosch JM, Lammers JW (2007). Diffusing capacity for nitric oxide: reference values and dependence on alveolar volume. Respir Med.

[16] Soriano JB, Lamprecht B, Ramirez AS, Martinez-Camblor P, Kaiser B, Alfageme I, Almagro P, Casanova C, Esteban C, Soler-Cataluna JJ (2015). Mortality prediction in chronic obstructive pulmonary disease comparing the GOLD 2007 and 2011 staging systems: a pooled analysis of individual patient data. Lancet Respir Med.

[17] Lucke T, Herrera R, Wacker M, Holle R, Biertz F, Nowak D, Huber RM, Sohler S, Vogelmeier C, Ficker JH (2016). Systematic analysis of self-reported comorbidities in large cohort studies - a novel stepwise approach by evaluation of medication. PLoS One.

[18] Sinha S, Guleria R, Misra A, Pandey RM, Yadav R, Tiwari S (2004). Pulmonary functions in patients with type 2 diabetes mellitus & correlation with anthropometry & microvascular complications. Indian J Med Res.

[19] Diaz AA, Pinto-Plata V, Hernandez C, Pena J, Ramos C, Diaz JC, Klaassen J, Patino CM, Saldias F, Diaz O (2015). Emphysema and DLCO predict a clinically important difference for 6MWD decline in COPD. Respir Med.

[20] S A, S M, P G, C R: Alveolar Gas Exchange and Pulmonary Functions in Patients with Type II Diabetes Mellitus. J Clin Diagn Res 2013, 7(9):1874–1877. 10.7860/JCDR/2013/6550.3339 PMC3809625 24179886

[21] Fontaine-Delaruelle C, Viart-Ferber C, Luyton C, Couraud S (2016). Lung function in patients with diabetes mellitus. Rev Pneumol Clin.

[22] van den Borst B, Gosker HR, Zeegers MP, Schols AM (2010). Pulmonary function in diabetes: a metaanalysis. Chest.

[23] Paneni F, Beckman JA, Creager MA, Cosentino F (2013). Diabetes and vascular disease: pathophysiology, clinical consequences, and medical therapy: part I. Eur Heart J.

[24] Hoeper MM, Meyer K, Rademacher J, Fuge J, Welte T, Olsson KM (2016). Diffusion capacity and mortality in patients with pulmonary hypertension due to heart failure with preserved ejection fraction. JACC Heart Fail.

[25] Kothari V, Galdo JA, Mathews ST (2016). Hypoglycemic agents and potential anti-inflammatory activity. J Inflamm Res.

[26] Isoda K, Young JL, Zirlik A, MacFarlane LA, Tsuboi N, Gerdes N, Schonbeck U, Libby P (2006). Metformin inhibits proinflammatory responses and nuclear factor-kappaB in human vascular wall cells. Arterioscler Thromb Vasc Biol.

[27] Hattori Y, Hattori K, Hayashi T (2015). Pleiotropic benefits of metformin: macrophage targeting its anti-inflammatory mechanisms. Diabetes.

[28] Wang XC, Gusdon AM, Liu H, Qu S (2014). Effects of glucagon-like peptide-1 receptor agonists on non-alcoholic fatty liver disease and inflammation. World J Gastroenterol.

[29] Ling MY, Ma ZY, Wang YY, Qi J, Liu L, Li L, Zhang Y (2013). Up-regulated ATP-sensitive potassium channels play a role in increased inflammation and plaque vulnerability in macrophages. Atherosclerosis.

[30] Shinjo T, Nakatsu Y, Iwashita M, Sano T, Sakoda H, Ishihara H, Kushiyama A, Fujishiro M, Fukushima T, Tsuchiya Y (2015). DPP-IV inhibitor anagliptin exerts anti-inflammatory effects on macrophages, adipocytes, and mouse livers by suppressing NF-kappaB activation. Am J Physiol Endocrinol Metab.

[31] Dandona P, Aljada A, Mohanty P, Ghanim H, Hamouda W, Assian E, Ahmad S (2001). Insulin inhibits intranuclear nuclear factor kappaB and stimulates IkappaB in mononuclear cells in obese subjects: evidence for an anti-inflammatory effect?. J Clin Endocrinol Metab.

[32] Wright JG, Christman JW (2003). The role of nuclear factor kappa B in the pathogenesis of pulmonary diseases: implications for therapy. Am J Respir Med.

[33] Hitchings AW, Lai D, Jones PW, Baker EH (2016). Metformin in severe exacerbations of chronic obstructive pulmonary disease: a randomised controlled trial. Thorax.

[34] Sexton P, Metcalf P, Kolbe J (2014). Respiratory effects of insulin sensitisation with metformin: a prospective observational study. COPD.

[35] Gogitidze Joy N, Hedrington MS, Briscoe VJ, Tate DB, Ertl AC, Davis SN (2010). Effects of acute hypoglycemia on inflammatory and pro-atherothrombotic biomarkers in individuals with type 1 diabetes and healthy individuals. Diabetes Care.

[36] Bedenis R, Price AH, Robertson CM, Morling JR, Frier BM, Strachan MW, Price JF (2014). Association between severe hypoglycemia, adverse macrovascular events, and inflammation in the Edinburgh Type 2 Diabetes Study. Diabetes Care.

